# Central airway pathology: clinic features, CT findings with pathologic and virtual endoscopy correlation

**DOI:** 10.1007/s13244-017-0545-6

**Published:** 2017-02-15

**Authors:** Daniel Barnes, José Gutiérrez Chacoff, Mariana Benegas, Rosario J. Perea, Teresa M. de Caralt, José Ramirez, Ivan Vollmer, Marcelo Sanchez

**Affiliations:** 1 Radiology Department, Hospital Clinic de Barcelona, Villarroel 170, 08036 Barcelona, Spain; 2 Radiology Department, Clínica Dávila de Santiago, Santiago, Chile; 3 Pathology Department, Hospital Clinic de Barcelona, Barcelona, Spain

**Keywords:** Respiratory tract diseases, Tracheal diseases, Tuberculosis, Relapsing polychondritis, Tracheobronchomegaly

## Abstract

**Objectives:**

To describe the imaging features of the central airway pathology, correlating the findings with those in pathology and virtual endoscopy. To propose a schematic and practical approach to reach diagnoses, placing strong emphasis on multidetector computed tomography (MDCT) findings.

**Methods:**

We reviewed our thoracic pathology database and the central airway pathology-related literature. Best cases were selected to illustrate the main features of each disease. MDCT was performed in all cases. Multiplanar and volume-rendering reconstructions were obtained when necessary. Virtual endoscopy was obtained from the CT with dedicated software.

**Results:**

Pathological conditions affecting the central airways are a heterogeneous group of diseases. Focal alterations include benign neoplasms, malignant neoplasms, and non-neoplastic conditions. Diffuse abnormalities are divided into those that produce dilation and those that produce stenosis and tracheobronchomalacia. Direct bronchoscopy (DB) visualises the mucosal layer and is an important diagnostic and therapeutic weapon. However, assessing the deep layers or the adjacent tissue is not possible. MDCT and post-processing techniques such as virtual bronchoscopy (VB) provide an excellent evaluation of the airway wall.

**Conclusion:**

This review presents the complete spectrum of the central airway pathology with its clinical, pathological and radiological features.

**Teaching points:**

• *Dividing diseases into diffuse and focal lesions helps narrow the differential diagnosis.*

• *Focal lesions with nodularity are more likely to correspond to tumours.*

• *Focal lesions with stenosis are more likely to correspond to inflammatory disease.*

• *Posterior wall involvement is the main feature in diffuse lesions with stenosis.*

## Introduction

Large-airway pathological conditions are a heterogeneous group of diseases that include focal and diffuse lesions. Although tracheobronchial neoplasms are uncommon, there is a high incidence of malignancy [1]. Furthermore, most of the benign neoplasms and inflammatory conditions are usually symptomatic and need treatment. Focal lesions may be subdivided into benign neoplasms (papilloma, hamartoma, and carcinoid), malignant neoplasms (squamous cell carcinoma, adenoid cystic carcinoma, other primary neoplasms such as lymphoma or haemangiopericytoma, and secondary malignancy), and non-neoplastic conditions (tuberculosis, post-intubation stenosis, idiopathic subglottic stenosis, post-inflammatory pseudotumour, trauma, and foreign body). Diffuse lesions can be classified into lesions with dilatation of the tracheobronchial lumen (Mounier-Kuhn syndrome and acquired tracheobronchomegaly), lesions with stenosis (rhinoscleromatosis, granulomatous bronchitis, amyloidosis, sarcoidosis, granulomatosis with polyangitis, relapsing polychondritis, osteochondroplastic tracheobronchopathy), and lesions with respiratory collapse (tracheobronchomalacia).

Multiple detector computed tomography (MDCT) plays a key role in the identification and characterisation of various large-airway diseases, and post-processing tools, such as virtual bronchoscopy, may improve the performance of the study [2]. An MDCT study of the airways needs axial thin sections (ideally 1 mm sections with 80% overlap), multiplanar reformations, minimum intensity projections, volume-rendering images, virtual bronchoscopy images, and sometimes dynamic studies with inspiration and espiration acquisition. The whole set of images and reformations is necessary to make an adequate interpretation of an airways CT study. Intravenous contrast is usually not necessary in benign pathology and is useful in cases of neoplasm.

We describe the pathological conditions that affect the trachea, its clinical characteristics and MDCT features, and propose a schematic diagnostic approach that allows directing the study.

### Focal lesions

#### Benign neoplasms

##### Papilloma

Papilloma represents an abnormal proliferation of the squamous epithelium in the respiratory tract, secondary to infection with human papilloma virus (HPV). Most of the cases correspond to juvenile onset form with upper respiratory tract infection. Tracheobronchial compromise is most common in the adult-onset form and involves just 5% of patients, generally between 20 and 40 years of age, infected by sexual transmission (oral contact), with a male:female ratio of 4:1 [3]. HPV-6 and HPV-11 are responsible for airway infections and have low risk of cancer compared to HPV-16 and HPV-18, which cause the majority of cervical cancers [4].

**Fig. 1:**
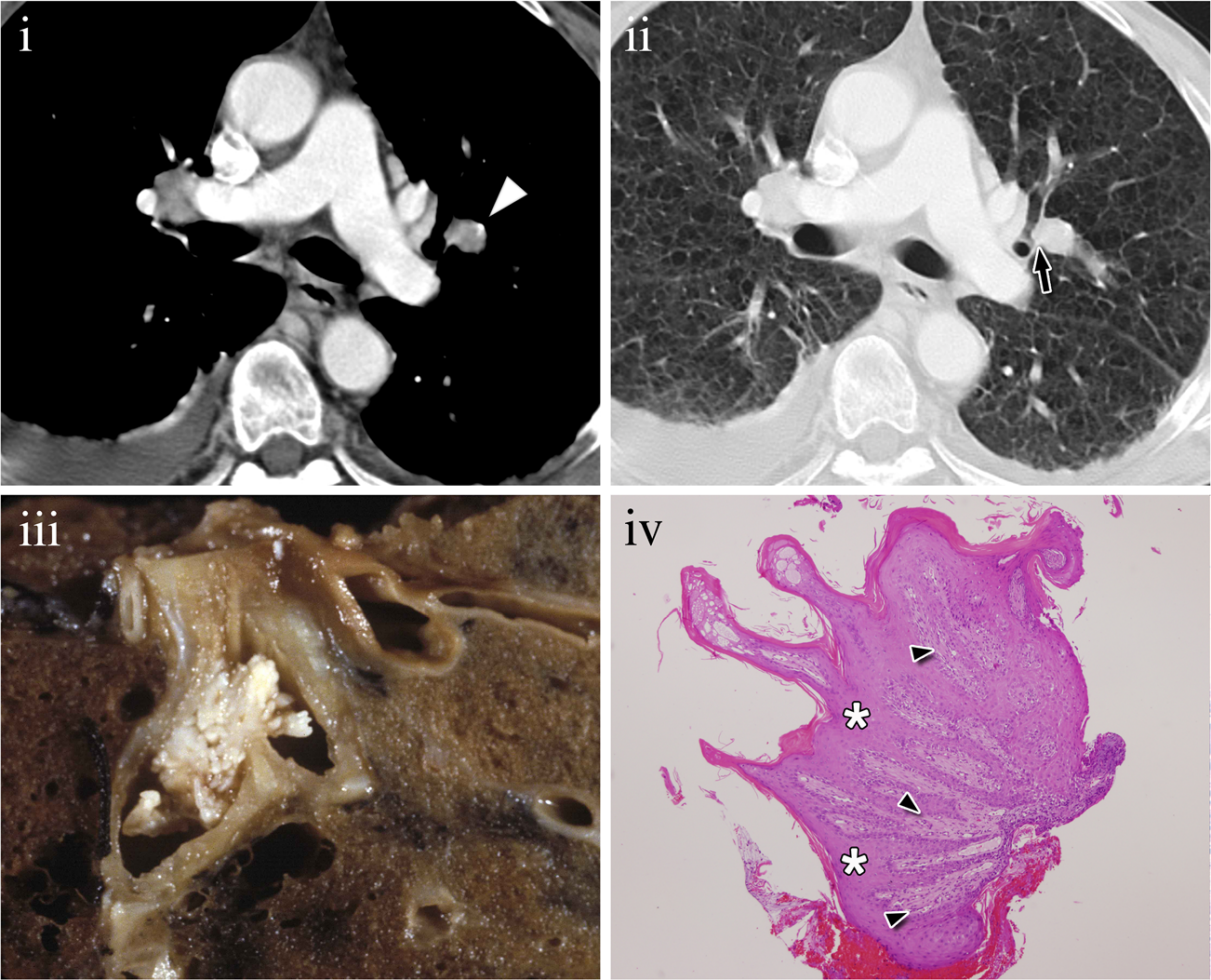
Endobronchial papilloma of a 50-year-old patient. (**i**) Contrast-enhanced CT scan of the chest in mediastinal window shows a mildly enhancing nodule (*arrow*). (**ii**) Lung window confirms its endobronchial origin (arrow). Diagnosis was made after resection. (**iii**) Photograph of the resected lung shows a polypoid irregular intrabronchial lesion. (**iv**) Photomicrograph (original magnification, ×2; haematoxylin-eosin stain) shows an exophytic lesion with benign keratinized squamous epithelium (stars) covering an irregular stroma (*arrowheads*)

MDCT shows nodules arising from the mucosal surface, with intraluminal growth (Fig. 1). Lesions may be unique or multiple and may cause atelectasis of the underlying lung, with symptoms of an obstructive pneumonia. One per cent of the patients have pulmonary spread, which manifests on CT as multiple pulmonary nodules with air trapping areas.

##### Hamartoma

Endobronchial hamartoma is a lesion with a true neoplastic origin, which represents 3–20% of all lung hamartomas [5]. The most frequent types of endobronchial hamartoma are chondromatous and lipoid [6]. There is also an extremely rare osteochondromatosis variety [7].

**Fig. 2:**
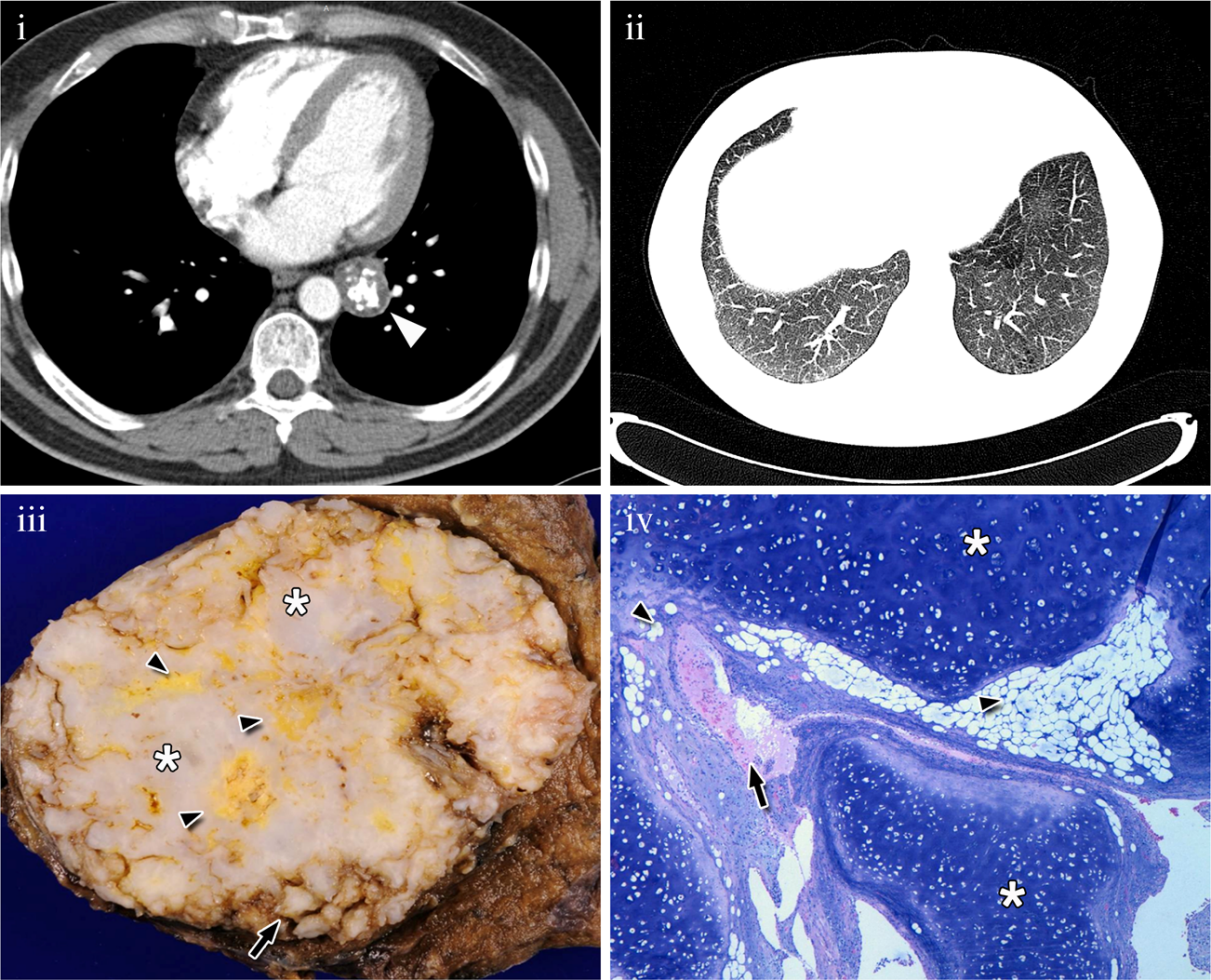
Hamartoma in a 62-year-old patient. (**i**) Contrast-enhanced CT scan showing a lesion (*arrowhead*) with popcorn-like calcification and fat-tissue attenuation, consistent with hamartoma. (**ii**) Endobronchial involvement produces air trapping in the anterobasal segment because of valve effect. (**iii**) Gross pathology specimen showing a multilobulated tumour lesion that combines adipose (*arrowheads*), cartilaginous (stars) and epithelial tissue invaginations (*arrow*) consistent with hamartoma. (**iv**) Same findings are shown in the photomicrograph (original magnification, 4×; haematoxylin-eosin stain)

The hamartoma is composed of chondroid cartilage, observed as a popcorn calcification within the lesion on CT, fat, represented by areas of low attenuation, and fibrous and epithelial tissue, both with soft-tissue attenuation [8]. The combination of fat attenuation areas and popcorn calcifications is considered diagnostic for hamartoma on CT [9] (Fig. 2). Bronchial hamartomas tend to have more fat than pulmonary hamartomas because the bronchial wall is rich in fat [6] and popcorn calcification helps to differentiate hamartomas from malignant tumours.

Unlike lung hamartomas, bronchial hamartomas are generally symptomatic because of bronchial obstruction or bleeding, manifested as obstructive pneumonia (with cough and/or dyspnoea) or haemoptysis [8, 9].

##### Carcinoid

The carcinoid is part of the spectrum of neuroendocrine cell neoplasms, and the respiratory tract is the second most common location (20–30% of all carcinoids), behind the gastrointestinal tract [10]. Most carcinoids compromise the central bronchus, and tracheal carcinoids are extremely rare [11].

Those tumours arise from Kulchitsky cells, and two different biological behaviours are recognised. The typical carcinoid has less than two mitoses per ten high-power fields (HPF), and no necrosis, whereas atypical carcinoids have from two to ten mitoses per HPF or necrosis [12]; 80–90% of bronchial carcinoids are typical [10].

Although carcinoids may secrete active substances, such as serotonin, carcinoid patients rarely have carcinoid syndrome [13], since excess serotonin is broken down by the liver. Characteristically, the patients with liver metastases (1–5% of the cases) may develop carcinoid syndrome because they lose the hepatic filter [14]. Other paraneoplasic presentations, such as Cushing syndrome, are seen in less than 2% of the cases. Up to 50% of the patients with carcinoid syndrome may develop carcinoid heart disease, consisting of right-side heart failure, secondary to valvulopathy [15].

**Fig. 3:**
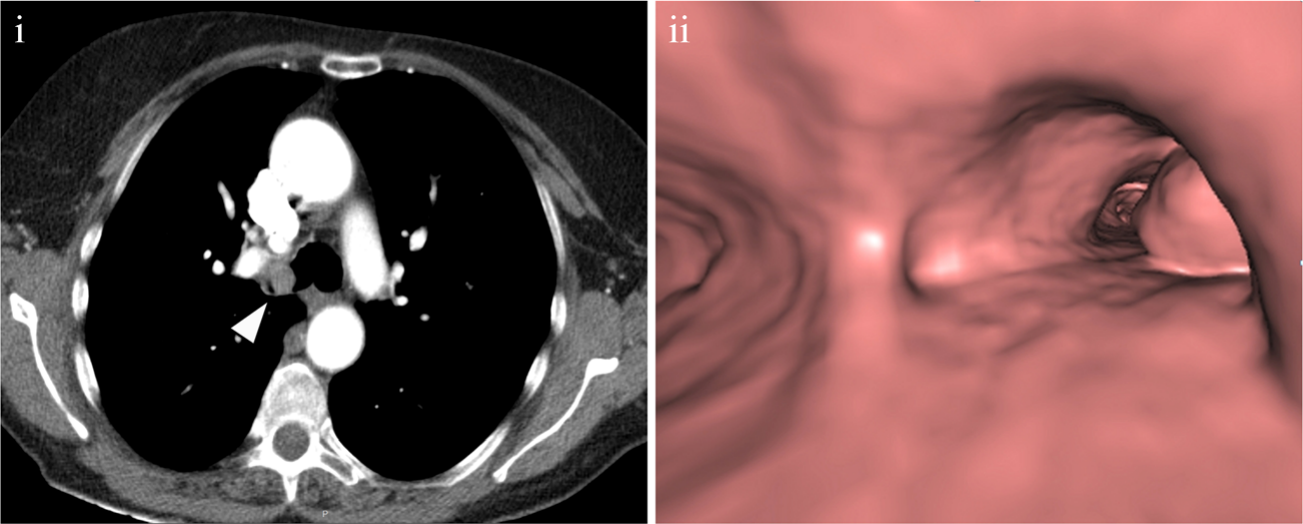
(**i**) Contrast-enhanced CT scan in a 55-year-old patient showing a nodule within the right main bronchus (*arrowhead*) with avid contrast enhancement, suggestive of a carcinoid tumour. After resection, pathological analysis (not shown) confirmed the diagnosis. (**ii**) Virtual bronchoscopy depicts the lesion

At CT, a carcinoid tumour in the tracheobronchial tree appears as a well-defined spherical or ovoid nodule with a slightly lobulated border, with important contrast enhancement [13, 16] (Fig. 3). Punctate or diffuse calcifications may be present in up to 30% [17]. Carcinoids tend to produce different degrees of bronchial obstruction, which are detected at CT as indirect signs, from air trapping or a mucoid bronchogram to lobar or segmental atelectasis [17]. There are cases in which the bronchial carcinoid presents as an “iceberg tumour”, with a small intraluminal lesion, undetectable even by direct bronchoscopy, and a large extraluminal component, easily detected by CT [10].

#### Malignant neoplasms

##### Squamous cell carcinoma (SCC)

SCC is the most common primary tracheobronchial tumour. It develops mainly in the 6th and 7th decades of life, being two to four times more common in men than in women, and it is strongly related to smoking [18].

Tracheobronchial SCC is histologically identical to lung SCC, and metachronous or synchronous lesions are common in the oropharynx, larynx, and lungs in up to 40% of the cases [18].

It is commonly infiltrative in nature, with exophytic or ulcerative lesions, which may produce haemoptysis. When the tumour produces stenosis larger than 50% of the airway diameter, it generates obstructive symptomatology, with cough, dyspnoea and wheezing.

**Fig. 4:**
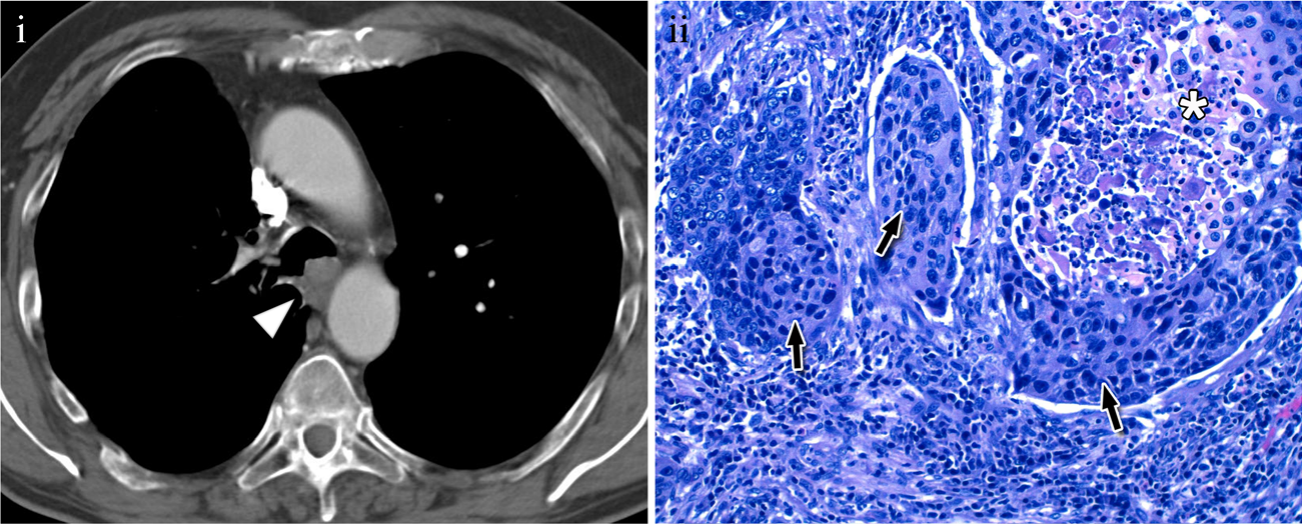
Squamous cell carcinoma in a 63-year-old patient, a heavy smoker. (**i**) Axial CT scan shows a nodular lesion (*arrowhead*) located on the tracheal carina with an endoluminal and extraluminal component. (**ii**) Photomicrograph (original magnification, ×20; haematoxylin-eosin stain) shows infiltrating groups of atypical squamous epithelial cells (*arrow*), with necrotising material in the centre of the epithelial nest (star)

CT imaging often shows a polypoid intraluminal mass with irregular, smooth, or lobulated contours in the lower third of the trachea [11, 13] (Fig. 4), but it can also be seen as an eccentric narrowing of the airway or as circumferential wall thickening. Mediastinal adenopathies or pulmonary metastases are present in about one-third of patients at the time of diagnosis [13].

##### Adenoid cystic carcinoma

This is the second most common tracheal malignancy after squamous cell carcinoma [13, 19], and it is the most common tumour of salivary glands in the large airways.

This tumour has no relation with smoking [20]. The patients are generally younger than 40 years of age, and there is no difference in gender distribution [19, 21].

Clinically, these tumours present symptoms related with obstruction, generally low grade, which may be confused with asthma or bronchitis [20]. Haemoptysis is less common than in squamous cell carcinoma [11].

**Fig. 5:**
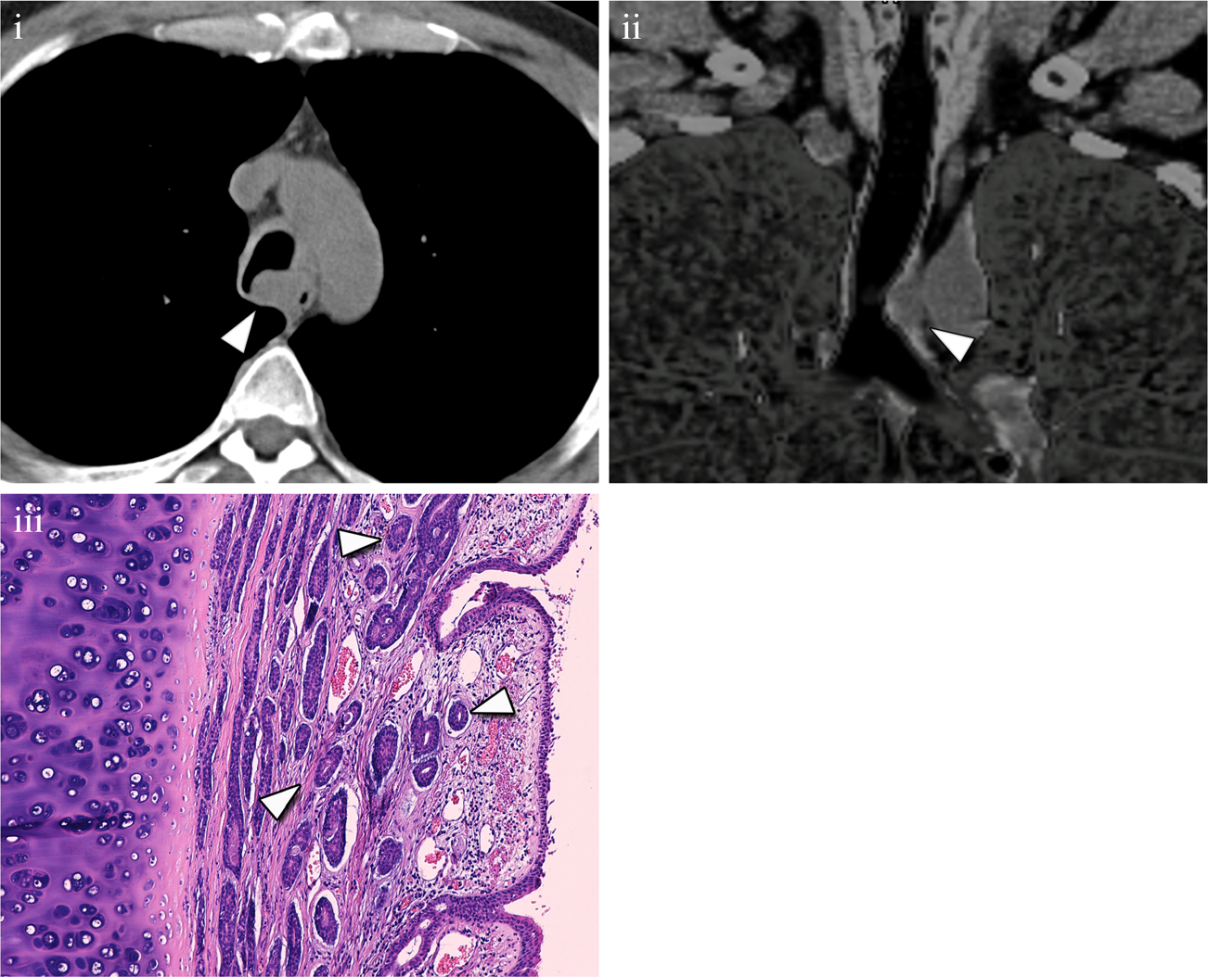
Cystic adenoid carcinoma in a 44-year-old patient, with no smoking history. (**i**) Axial MDCT (*arrowhead*) shows an endoluminal mass with an extraluminal solid component that deforms the left side of the tracheal wall. (**ii**) Coronal MDCT reconstruction demonstrates that the longitudinal axis of the lesion is greater than the axial axis. These are the typical findings in a cystic adenoid carcinoma. After resection, pathological analysis confirmed the diagnosis. (**iii**) Photomicrograph (original magnification, ×4; haematoxylin-eosin stain) shows an extensive interstitial infiltration by groups of tumoral cells with pseudo-glandular pattern (*arrowheads*)

Adenoid cystic carcinoma arises most frequently in the lower trachea and main bronchi and has predominantly submucosal extension [19], appearing as a lesion with a smooth contour and intact mucosa at direct bronchoscopy [22, 23]. CT shows a smooth mass with endoluminal and extraluminal growth, and soft tissue attenuation, which usually involves more than 180° of the airway circumference and often encircles the lumen (Fig. 5) [20]. The longitudinal axis of the tumour is classically greater than its axial extent [13, 24]. Therefore, the use of multiplanar reconstructions is very useful for not underestimating the tumour size [25].

##### Other neoplastic malignancies

**Fig. 6:**
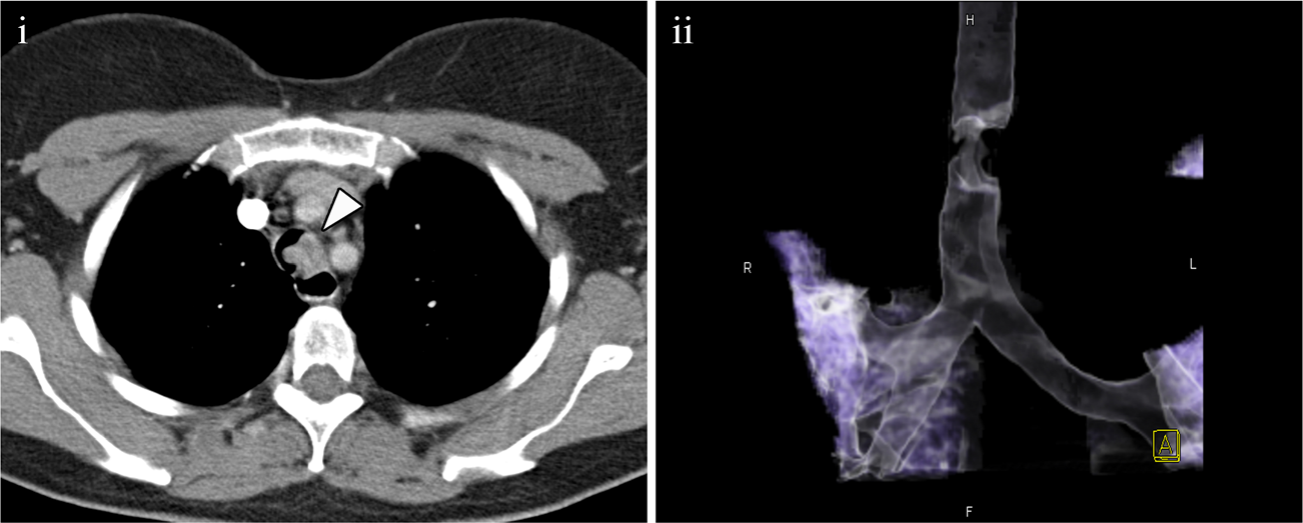
Haemangiopericytoma. (**i**) Axial MDCT showing an intratracheal mass (*arrowhead*) with irregular margins, with no specific features. (**ii**) Volume-rendering reconstruction showing the important irregular stenosis. After surgery and pathological analysis, the diagnosis of haemangiopericytoma was made

**Fig. 7:**
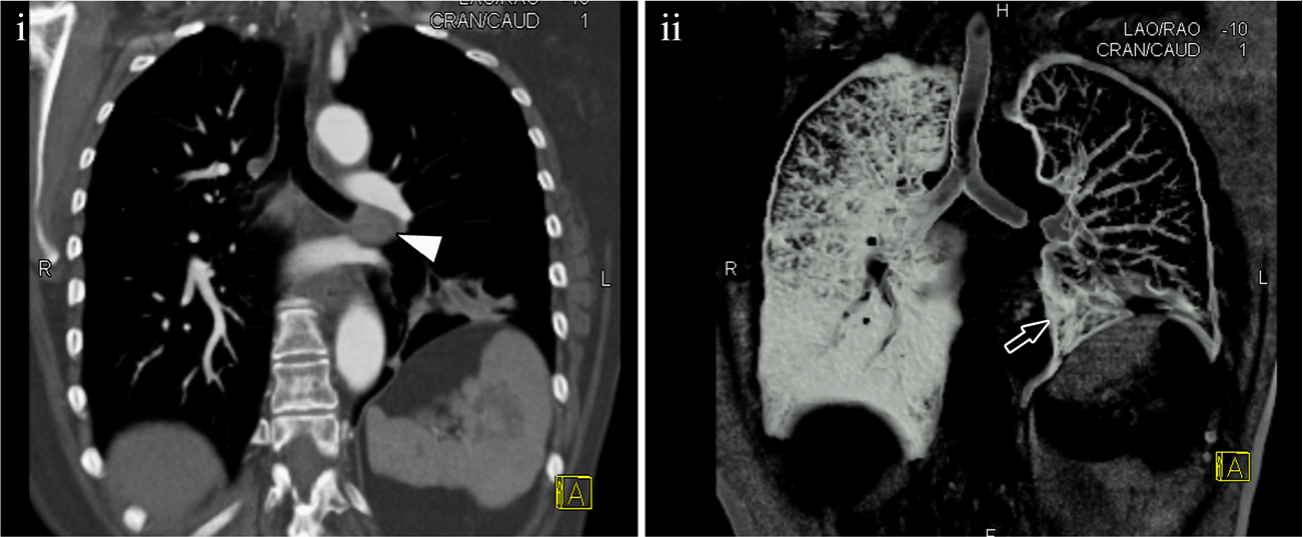
Lymphoma. (**i**) Coronal multiplanar reconstruction showing an endobronchial enhancing lesion in the left main bronchus (*arrowhead*) that produces an almost complete occlusion of the bronchus. (**ii**) Volume-rendering reconstruction better depicting the occlusion of the main left bronchus. Note the air trapping at the upper lobe of the left lung and the loss of volume of the inferior lobe (*arrow*). Histological analysis (not shown) yielded the diagnosis of lymphoma

There is a group of infrequent malignant neoplasms, such as haemangiopericytoma (Fig. 6) or lymphoma (Fig. 7), whose characteristics are not specific. Their diagnosis is not suspected with imaging, and histological studies are required.

##### Secondary malignancy

**Fig. 8:**
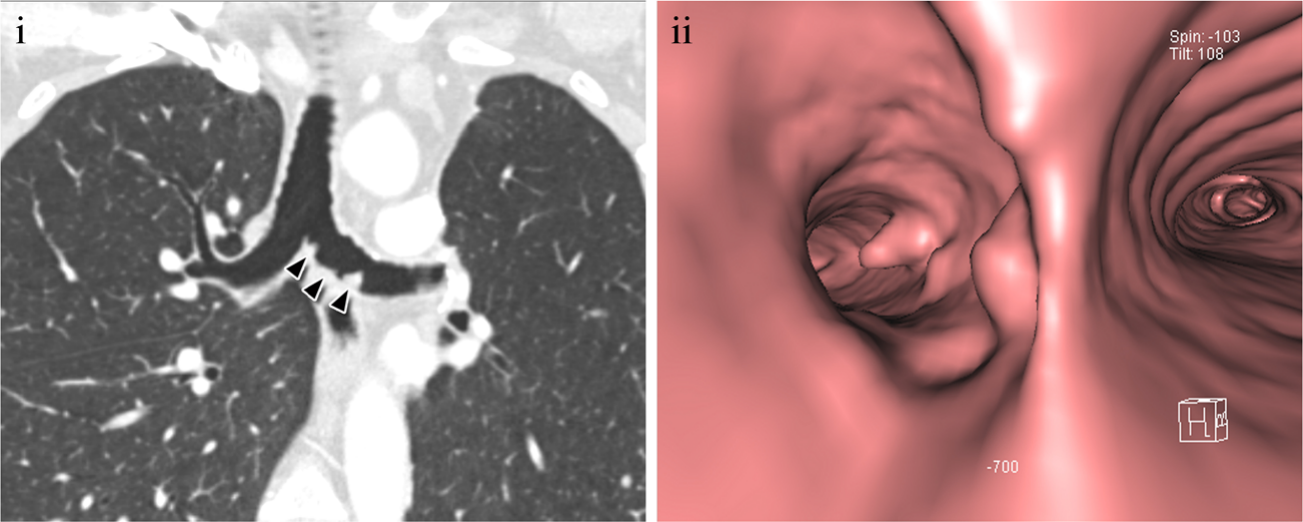
A 58-year-old patient with metastatic colon cancer. (**i**) Multiplanar coronal reconstruction showed a newly appeared small nodular lesion within the left bronchus wall (*arrowheads*), highly suggestive of progression of the disease. (**ii**) Virtual bronchoscopy showed more irregularities in the bronchus wall

Metastatic compromise of the tracheobronchial wall is infrequent. Direct invasion usually comes from lung, thyroid, oesophageal, or laryngeal neoplasm and may cause obstruction or fistula. Haematogenous spread, even more rare, may originate from lung, breast, colorectal, renal, uterine, and skin cancer [26]. On CT, they are undistinguishable from primary tumours and may present as a solitary tumour or multiple lesions. The possibility of endotracheal or endobronchial metastasis should be considered if the patient has a history of malignancy in other organs (Fig. 8). To be considered secondary, the lesion must be histopathologically identical to the previously documented primary tumours [27].

#### Non-neoplastic lesions

##### Tracheobronchial tuberculosis

Some degree of airway stenosis occurs in up to 90% of patients with endobronchial tuberculosis despite appropriate therapy [28]. However, tracheobronchial tuberculosis has been reported in 10 to 38.8% of patients with pulmonary parenchymal tuberculosis through bronchoscopic examination [29]. The development of the disease may be secondary to direct infection of the mucosa, submucosal lymphatic spread, or direct extension from infected adenopathy [24].

**Fig. 9:**
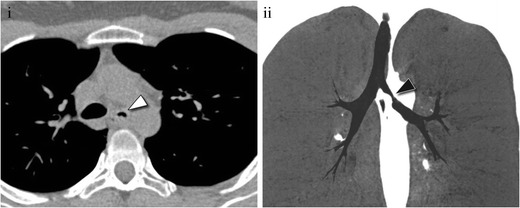
Tuberculosis in the left main bronchus of a 31-year-old female. (**i**) Axial MDCT scan shows a significant stenosis of the main left bronchus, without the presence of a mass. (**ii**) Coronal MinIP reconstruction shows a short area of stenosis located at the left main bronchus (*arrow*)

Most stenoses are believed to be caused by infectious necrosis and ulceration of the bronchial mucosa that leads to granulation and scarring [23]. Active lung infection is not required to develop airway narrowing. Infectious necrosis and ulceration of the bronchial mucosa lead to granulomatous scarring, which causes a fibrotic lesion with secondary stenosis [28, 30]. In active tuberculosis, MDCT may show irregular wall narrowing, with heterogeneous contrast enhancement (Fig. 9). The fibrotic lesion is seen as a circumferential smooth stenosis, with minimal wall thickening [28]. The disease tends to be multifocal, usually with a normal airway between structures [11], and it usually affects the left main bronchus and distal trachea [30].

##### Post-intubation stenosis

Luminal stenosis of the upper trachea can occur in patients with a history of tracheal intubation. High pressure in the tube balloon for prolonged periods may produce necrosis of the tracheal mucosa, leading the development of scar fibrosis, which produces luminal stenosis [16]. The phenomenon may be seen with orotracheal and tracheostomy tubes and is the most common cause of acquired tracheal stenosis.

The use of low pressure cuffs has reduced the incidence of tracheal stenosis post-intubation to less than 1% [28].

**Fig. 10:**
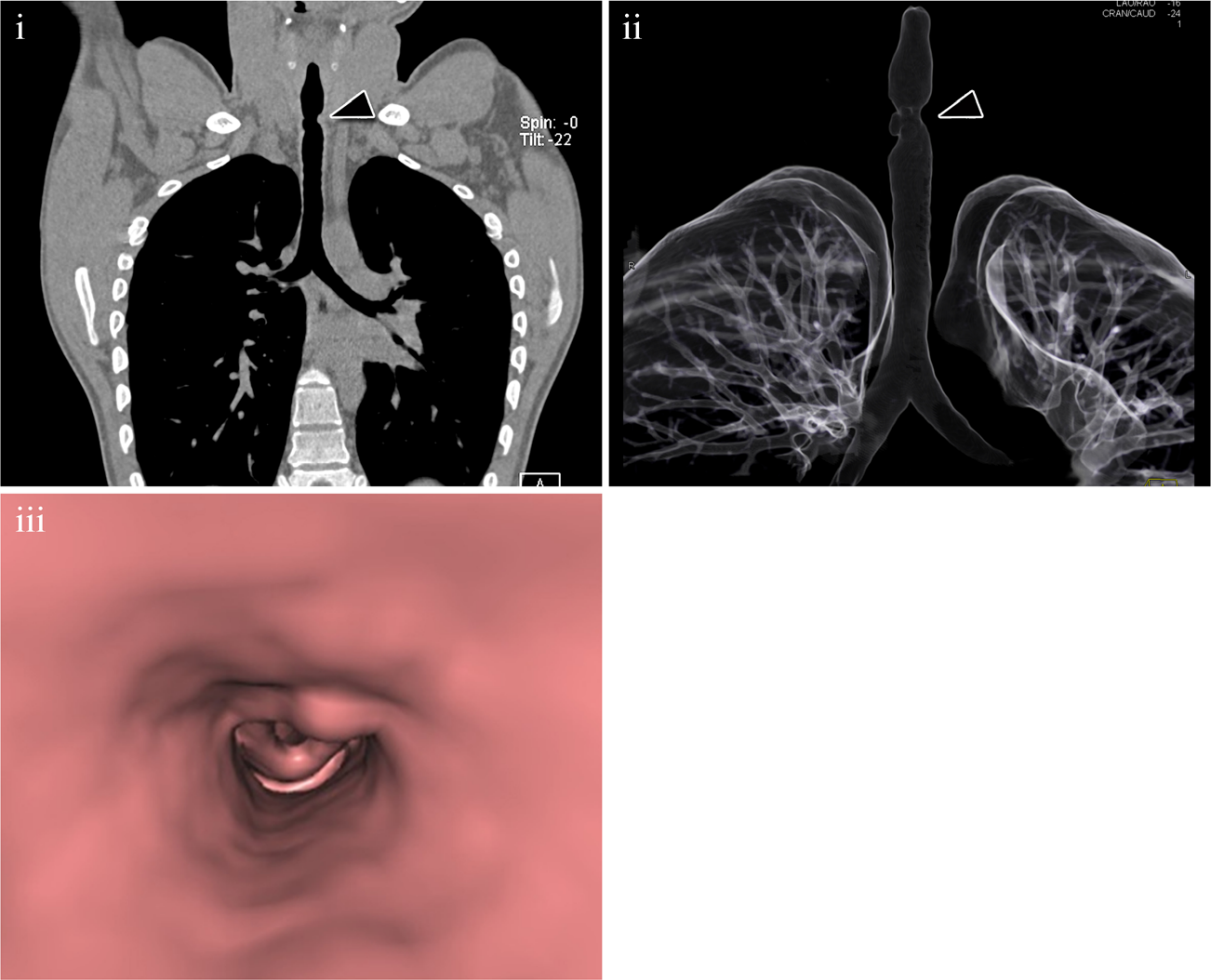
A 20-year-old patient developed dyspnoea after a long period of intubation. MDCT was performed. (**i**) Multiplanar coronal reconstruction and (**ii**) volume rendering showing stenosis in the upper trachea (*arrowhead*). Planimetry, not shown, yielded stenosis of 54%. (**iii**) Virtual bronchoscopy showing the lesion

MDCT has 100% specificity for the diagnosis of post-intubation stenosis, characteristically showing a concentric narrowing with an hourglass shape, located in the subglottic area and measuring less than 2 cm [16, 28, 31] (Fig. 10).

##### Idiopathic tracheal stenosis

Rare inflammatory cicatricial stenosis of the upper trachea, without known cause. The disease affects almost exclusively females, which some authors explain by the absence of receptors from the sites of stenosis, allowing the increased release of fibroblast growth factor [32]. Another factor that has been involved is gastroesophageal reflux, and although its role is still undetermined, improvement in idiopathic tracheal stenosis with treatment for reflux has been reported [33].

Most patients indicate having had symptoms for 1 to 3 years, presenting progressive dyspnoea, stridor, wheezing, and dry cough, and they have usually been treated for bronchitis before [34].

**Fig. 11:**
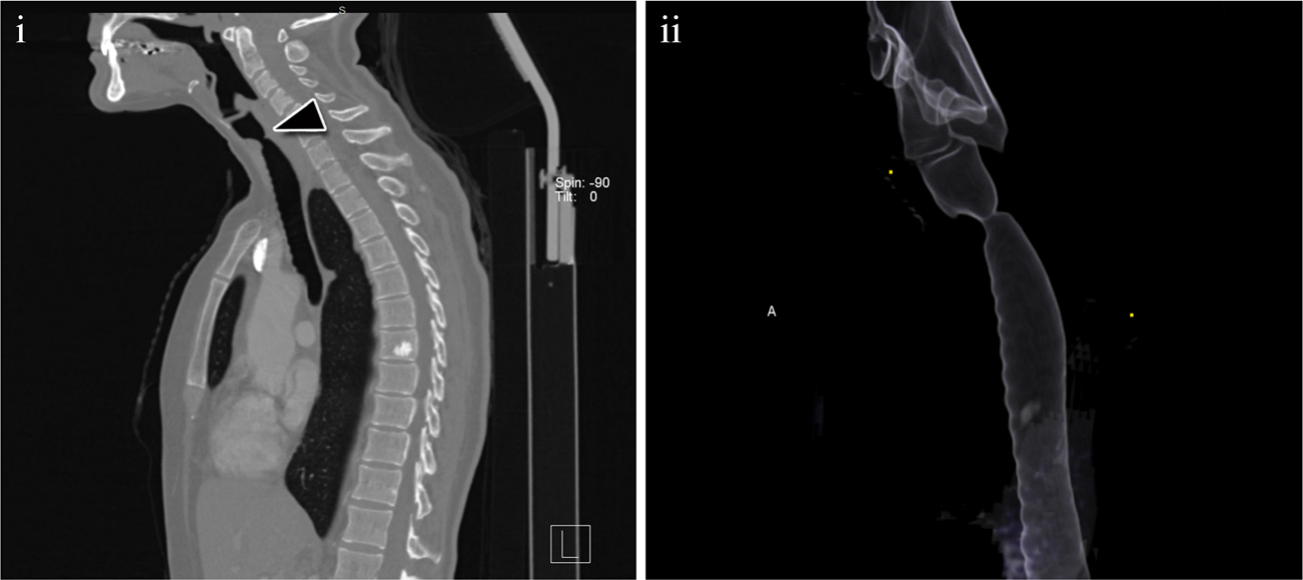
A 51-year-old female with a long history of progressive dyspnoea and stridor. (**i**) Multiplanar sagittal reconstruction showing a severe subglottic stenosis (*arrowhead*). (**ii**) Lateral view of a volume-rendering reconstruction of the same study. No other causes were found, and an exclusion diagnosis of idiopathic stenosis was made

To establish the diagnosis of idiopathic tracheal stenosis other causes of tracheal stenosis must be ruled out [34]. Bronchoscopy, spirometry, and MDCT are important for the diagnosis and management of the disease. MDCT shows focal stenosis in the subglottic trachea, very similar to post-intubation stenosis [35] (Fig. 11).

##### Inflammatory pseudotumour

This is a rare inflammatory mass composed of a polymorphous inflammatory cell infiltrate and variable amounts of fibrosis, necrosis, granulomatous reaction, and myofibroblastic spindle cells [36], which may mimic, clinically and radiologically, a neoplastic lesion.

Pseudotumour has been associated with IgG4-related sclerosing disease and with other inflammatory states such as trauma, surgery, and immune alterations [37].

**Fig. 12:**
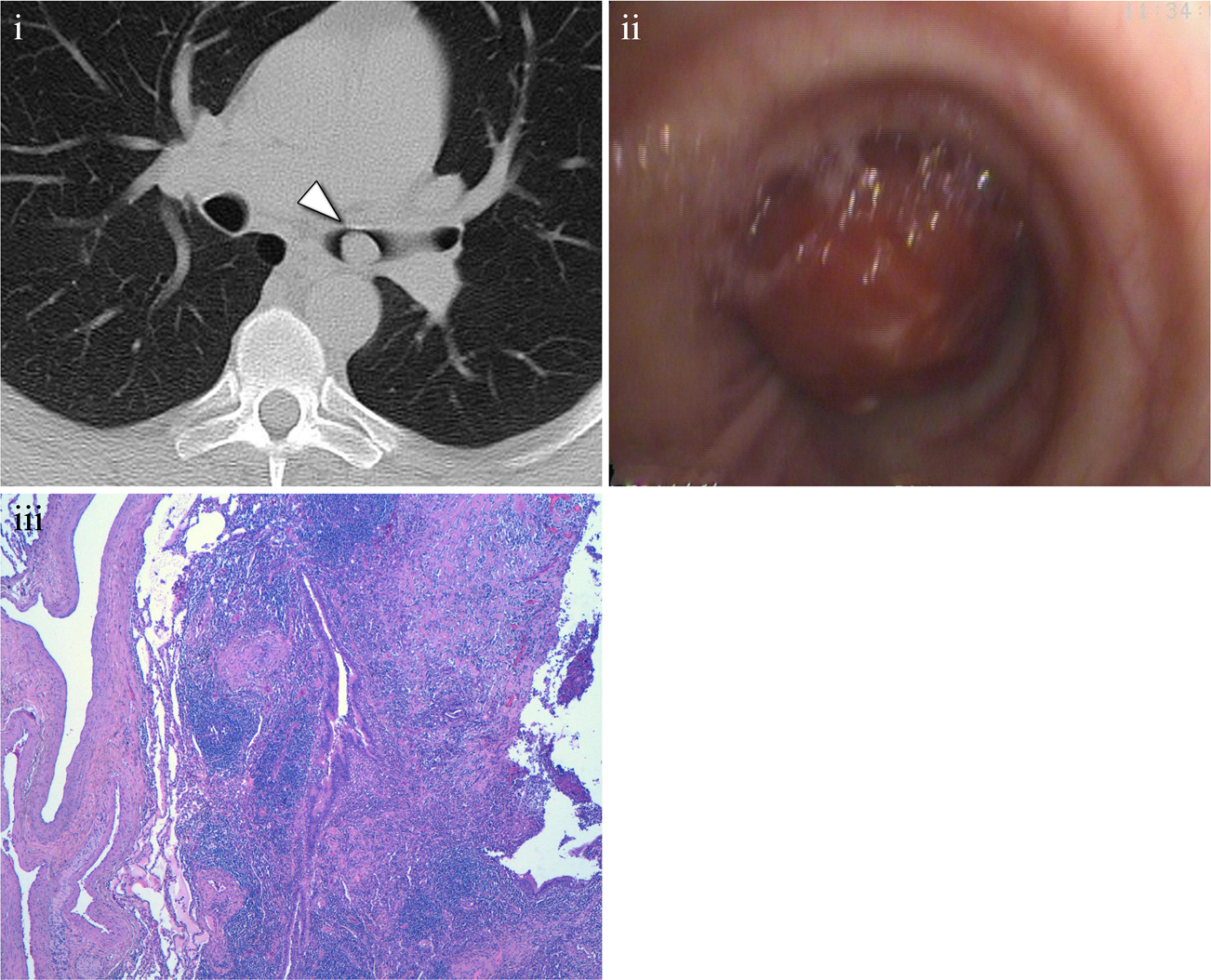
Inflammatory pseudotumour in the left main bronchus of a 50-year-old male, with a history of cough and wheeze for about 6 months. (**i**) Axial MDCT scan in the pulmonary window, showing a partially occlusive endobronchial nodule, located on the left main bronchus. (**ii**) Bronchoscopic image shows a hypervascular lesion, obstructing 95% of the bronchial lumen of the left main bronchus. Final diagnosis was made after resection. (**iii**) Photomicrograph (original magnification, ×4; haematoxylin-eosin stain) demonstrates an inflammatory infiltrate, composed mainly of myofibroblasts, characteristic of inflammatory pseudotumour

Patients can be asymptomatic or present a cough, chest pain, fever, dyspnoea, haemoptysis, and obstructive symptoms [38]. MDCT generally shows a rounded, well-defined endoluminal lesion on the trachea or main bronchi, with smooth contours that may have calcifications [36, 38], which enhances with the use of contrast, with a tendency to increase in delayed phases due to the fibrotic nature of the lesion (Fig. 12). An invasive behaviour is extremely rare.

The final diagnosis is made by bronchoscopic biopsy. Once the diagnostic has been confirmed, the elective treatment is endoscopic resection [38], with the exception of cases with transmural compromise, which must be resolved surgically.

##### Foreign body aspiration

Foreign body aspiration into the tracheobronchial tree is common and usually self-inflicted in children. In adults, however, it is rare and generally related to an iatrogenic or traumatic event [39]. Food and broken fragments of teeth are the most common foreign bodies aspirated, which tend to lodge in the right or left main bronchus with a similar frequency [40].

Penetration syndrome is the most common clinical presentation of foreign body aspiration and consists of sudden onset of choking, intractable cough, and vomiting, which may or may not be associated with wheezing, dyspnoea, and fever [41]. However, the clinical presentation varies, depending of the size of the foreign body, from the absence of symptoms to immediate asphyxiation and death when a large foreign body obstructs the trachea [42].

Chest radiography has low sensitivity for the diagnosis of foreign body aspiration, especially when the object is radiolucent, detecting 5–15% of cases [42].

CT is able to identify the level of obstruction and show the endobronchial foreign body (even low density objects), and it also allows the recognition of post-obstructive atelectases and air trapping areas. Multiplanar reconstructions and virtual bronchoscopy are very useful in the anatomic evaluation and planning of the extraction of the foreign body.

##### Tracheobronchial trauma

Tracheobronchial injuries are uncommon and include lacerations due to penetrating trauma and ruptures from blunt airway injury, particularly when the glottis is closed.

Blunt tracheobronchial injuries are generally located at about 2.5 cm of the carina, and they are the result of compression of the airway against the closed glottis [43].

**Fig. 13:**
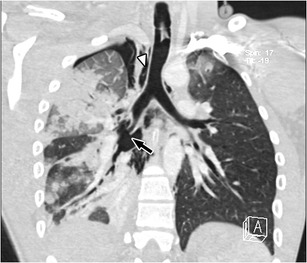
A 17-year-old male after a motorbike crash. MDCT scan and multiplanar coronal reconstruction showing complete rupture of the inferior lobe of the bronchus (*arrow*) causing pneumomediastinum (*arrowhead*) and pneumothorax. Areas of consolidation due to contusion are also seen in the upper as well as inferior lobes

Bronchial lacerations have a parallel direction to the bronchial cartilaginous ring [43] and commonly cause pneumomediastinum and pneumothorax. (Fig. 13) A pneumothorax refractory to evacuation by a correctly placed thorax tube also suggests bronchial rupture [44]. Complete transverse rupture may manifest by posterolateral displacement of the lung on the supine CT, also named the “fallen lung sign” [45].

Tracheal lacerations are less common and typically follow a vertical direction on the junction line of the cartilaginous and membranous portions of the tracheal wall. The presence of cervical subcutaneous emphysema and pneumomediastinum suggests a tracheal lesion. CT identifies the point of laceration in 70–100% of cases [46].

Bronchoscopy allows confirming the diagnosis and suturing the gap in some cases. However, surgical repair is required to prevent complications, such as infections and airway stenosis [44].

### Diffuse lesions

#### Mounier-Kuhn syndrome

Congenital tracheobronchomegaly is defined as significant dilation of the large airway, affecting the trachea and bronchi up to the fourth branch [45], secondary to severe atrophy of the longitudinal elastic fibres and thinning of the muscularis mucosa of the affected segments [16].

Typically, the diagnosis is made in males between 20 and 40 years of age with a history of recurrent pulmonary infections. Although most of the cases are sporadic, a familial susceptibility exists, and the syndrome has been associated with connective-tissue diseases, such as Ehlers-Danlos in adults or cutis laxa in children [46].

**Fig. 14:**
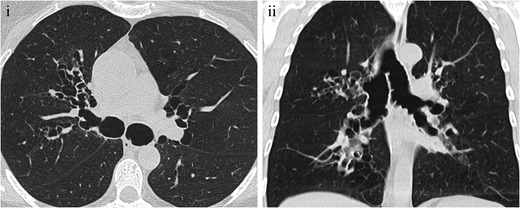
Mounier-Kuhn syndrome in a 45-year-old patient with a long history of lower respiratory tract infections. (**i**, **ii**) Axial and coronal MDCT scan, showing significant tracheobronchial dilatation and central bronchiectasis, both typical findings of Mounier-Kuhn syndrome

CT shows diffuse and symmetrical dilatation of the large airway, generally a tracheal diameter greater than 3 cm and main bronchi diameter greater than 2.4 cm, associated with central bronchiectasis (Fig. 14). Expiratory collapse is common, and the presence of tracheal diverticula gives the trachea a corrugated appearance [45].

#### Acquired tracheobronchomegaly

Tracheobronchial dilatation secondary to destruction of the muscular wall of the trachea and the main bronchi, as a tuberculosis sequela.

**Fig. 15:**
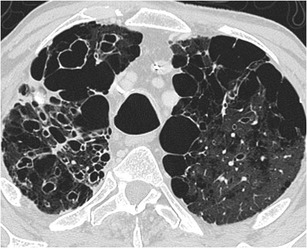
Acquired tracheobronchomegaly in a 72-year-old patient with a history of treated tuberculosis. (**i**) Axial MDCT shows tracheal dilatation, bronchiectasis, and tuberculosis sequelae, with a dense band and a calcification in the right superior lobe

Tracheal and proximal bronchi dilatation associated with pulmonary tuberculosis sequelae at CT and/or history of treated tuberculosis strongly suggests the diagnosis (Fig. 15).

#### Rhinoscleromatosis

Rhinoscleroma is a chronic, slowly progressive inflammatory disease of the upper respiratory tract, secondary to **Klebsiella rhinoscleromatis** infection [47]. Rhinoscleroma is found predominantly in rural areas with poor socioeconomic conditions, where infection is facilitated by crowding and poor hygiene and malnutrition.

The establishment of the disease is related to disabled macrophages, which allow bacterial multiplication within them, producing an ineffective delayed hypersensitivity response.

Scleroma primarily affects the nasal cavity (95–100%), but the nasopharynx (18–43%), oropharynx (13–35%), larynx (15–40%), trachea (12%), and bronchi (2–7%) can also be involved [48, 49].

The disease tends to progress slowly in periods of remission and relapse through three overlapping stages: the rhinitic stage, granulomatous stage with plasma cells and Mikulicz cells (histiocytes containing **K. rhinoscleromatis bacilli**) and fibrotic stage, characterised by scar tissue [28, 50]. A positive **K. rhinoscleromatis bacilli** culture is diagnostic for the disease but occurs in less than 60% of cases [51].

**Fig. 16:**
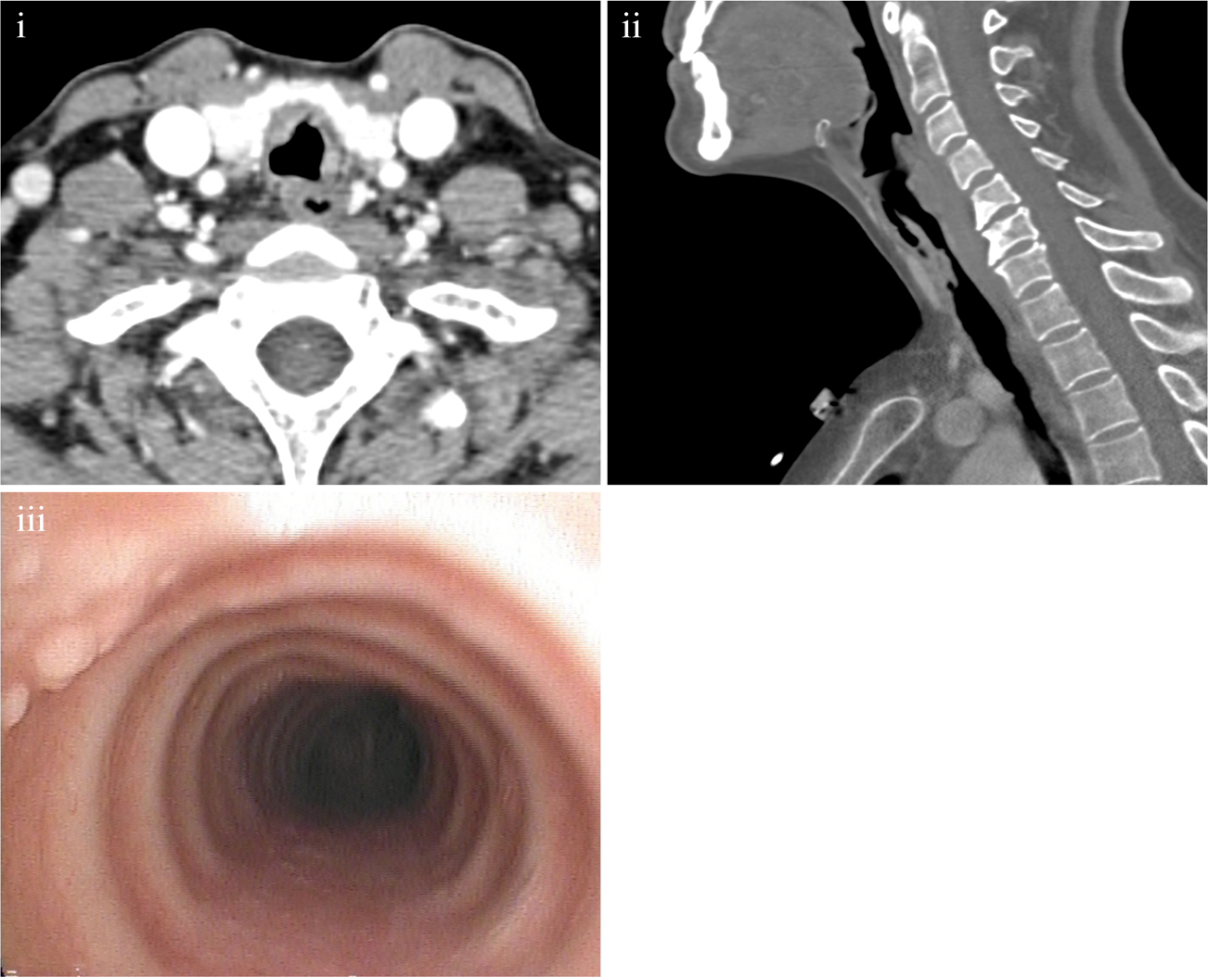
Tracheal rhinoscleroma in a 47-year-old male. (**i**) Axial MDCT scan shows a concentric irregular thickening of the trachea wall without evident calcifications. (**ii**) Sagittal MDCT scan demonstrates diffuse irregular narrowing of the tracheal wall, including the posterior. (**iii**) Bronchoscopy shows nodular plaques on the mucosal surface of the trachea

Tracheal rhinoscleroma is usually seen in continuity with laryngeal scleroma [49] and detected in the fibrotic stage, generally affecting the subglottic area. CT shows nodular concentric narrowing of the trachea, which may extend to the main bronchi. The lesions have soft tissue behaviour, classically without calcifications (Fig. 16) [28].

#### Granulomatous bronchitis

Granulomatous bronchitis is a cicatricial condition, secondary to a non-tuberculous infection sequela. Usually it is asymptomatic, but it may predispose the patient to pulmonary infections.

At MDCT, granulomatous bronchitis presents as central bronchiectasis in a focal area with wall thickening and diffuse wall calcifications and normally corresponds to an incidental finding.

#### Amyloidosis

Amyloidosis is defined as abnormal extracellular deposits of amyloid protein, which may be idiopathic or associated with inflammatory, hereditary, or neoplastic disorders [52].

Thoracic disease is an unusual manifestation of primary amyloidosis and may present as tracheobronchial deposits, diffuse interstitial infiltration of the lungs, and pulmonary nodules [28].

Tracheobronchial amyloidosis is the most common presentation form of thoracic amyloidosis, and it is characterised by the deposition of amyloid material as submucosal plaques and/or polypoid tumours in the airways [53], which may be localised, diffuse, or multifocal.

Patients are occasionally asymptomatic but often present with dyspnoea, wheezing, cough, haemoptysis, or recurrent pneumonia. The symptoms are usually nonspecific, with recurrent pulmonary infections or mimicking bronchial asthma [8].

**Fig. 17:**
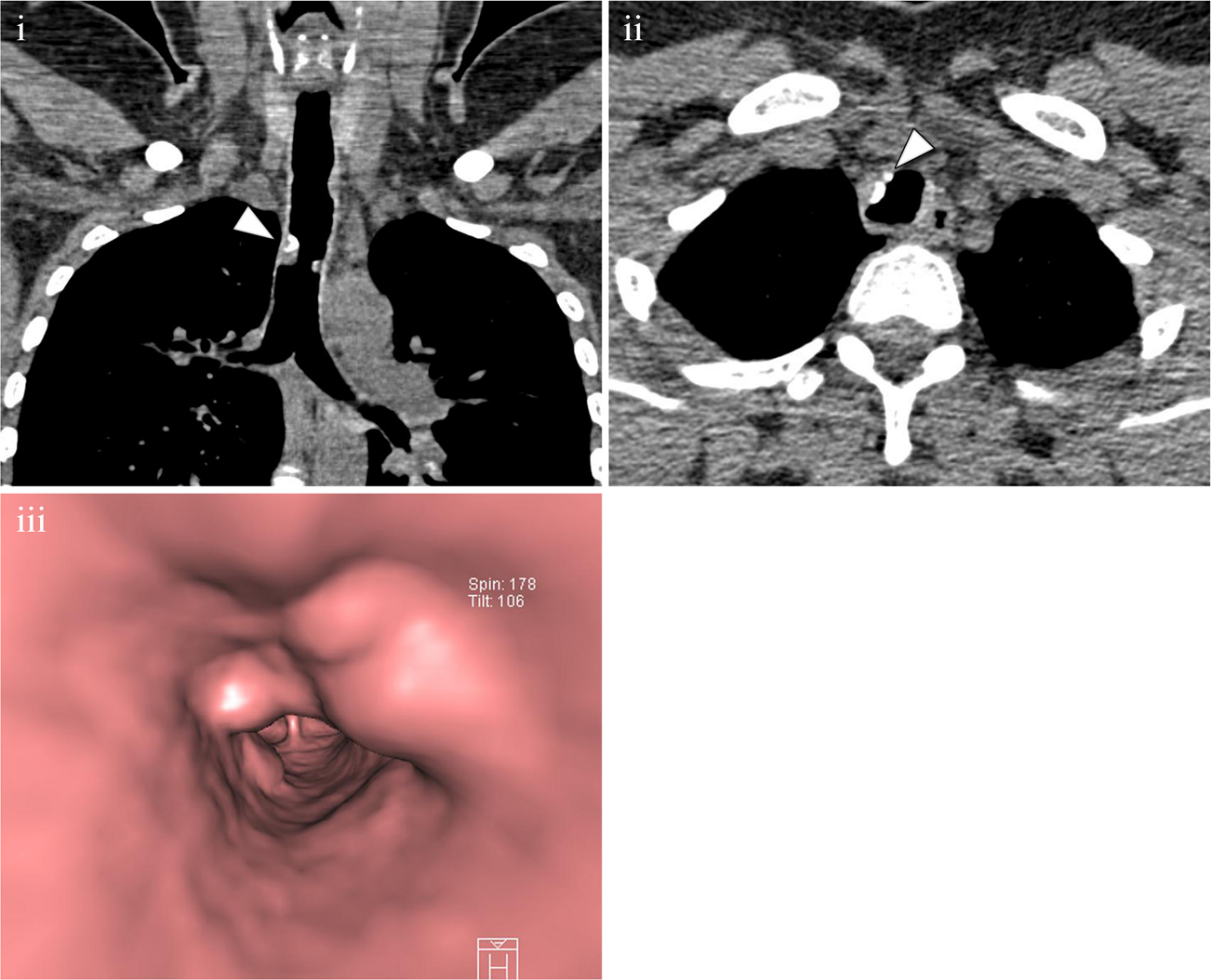
Tracheal amyloidosis in a patient with a history of pulmonary amyloidosis and episodic dyspnoea. (**i**) Coronal MDCT scan shows tracheal nodules protruding into the tracheal lumen, which has calcifications. (**ii**) Axial MDCT scan demonstrates the nodular affectation and the compromised posterior tracheal wall. (**iii**) Virtual endoscopy shows diffuse nodular narrowing of the tracheal lumen

MDCT shows irregular narrowing of the lumen with a nodular surface. The lesions may extend to the main bronchi, and they usually show calcifications and ossifications (Fig. 17). Furthermore, the lesions do not affect the posterior wall of the airway, which is an important point in the differential diagnosis [28].

#### Granulomatosis with polyangitis

Granulomatosis with polyangitis is an idiopathic necrotising granulomatous vasculitis, which is capable of affecting all organs but has a predilection for the upper respiratory tract, lungs, and kidneys [54].

The presentation forms vary from localised granulomatous disease of the respiratory tract to a medium-sized vessel vasculitis, with multiorgan compromise, predominantly renal and pulmonary. Therefore, clinical presentation is usually not specific and includes upper and lower respiratory tract symptoms, weight loss, and fever, which lead to a delay in diagnosis. Some patients may present with respiratory or renal failure [55]. The serum level of antineutrophil cytoplasmic antibodies directed against proteinase 3 is elevated in up to 90% of patients with granulomatosis with polyangitis, and it is considered an inflammatory activity marker. However, positivity is not conclusive for the diagnosis and negative test results do not exclude the disease. Biopsy still remains the standard means of diagnosis [56].

The tracheobronchial tree is the second most commonly affected area in the thorax in granulomatosis with polyangitis, involving 15 to 55% of the patients, generally in the context of multisystemic disease, and it is more common in females under 30 years of age [57].

**Fig. 18:**
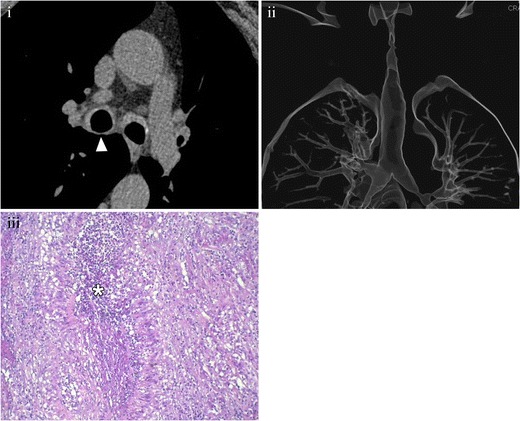
Tracheobronchial granulomatosis with polyangitis in a 57-year-old patient with a long history of multisystemic disease. (**i**) Axial MDCT scan shows a diffuse thickening of the bronchial wall, including the posterior wall, which helps to differentiate it from polychondritis. (**ii**) Coronal VRT reconstruction demonstrates stenosis on the subglottic area and left main bronchus. (**iii**) Photomicrograph (original magnification, ×20; haematoxylin-eosin stain) shows a necrotising granulomatous lesion with focal vascular destruction. The shape of the granuloma is elongated and the material in the necrotic centre has abundant nuclear debris

CT shows focal, multifocal, or diffuse thickening (nodular or smooth) of the tracheobronchial walls involving the posterior membrane, without the presence of calcifications [45] (Fig. 18). Circumferential compromise of the subglottic area of the trachea is the most frequent airway manifestation of granulomatosis with polyangitis and leads to stenosis in up to 25% of the patients [58]. Bronchial stenosis is just seen in 18% of the cases [58] and may produce atelectasis and bronchiectasis. Pulmonary manifestations (ground-glass opacities, consolidations, and solid or cavitated nodules) may also be present and are useful for the diagnosis [11].

#### Sarcoidosis

Sarcoidosis is a systemic granulomatous disease without a known aetiology, characterised histologically by the infiltration of affected tissue by non-caseating granulomatous inflammation. Although pulmonary and mediastinal involvement are the most common manifestation of sarcoidosis, large airway disease is infrequent, affecting 1–3% of the patients, mainly in the upper trachea. The distal part of the trachea and main bronchi are less affected [59].

There are two forms of central airway affectation, the extrinsic compression from hilar or mediastinal adenopathies and the wall affectation secondary to the formation within the mucosa and submucosa [28].

**Fig. 19:**
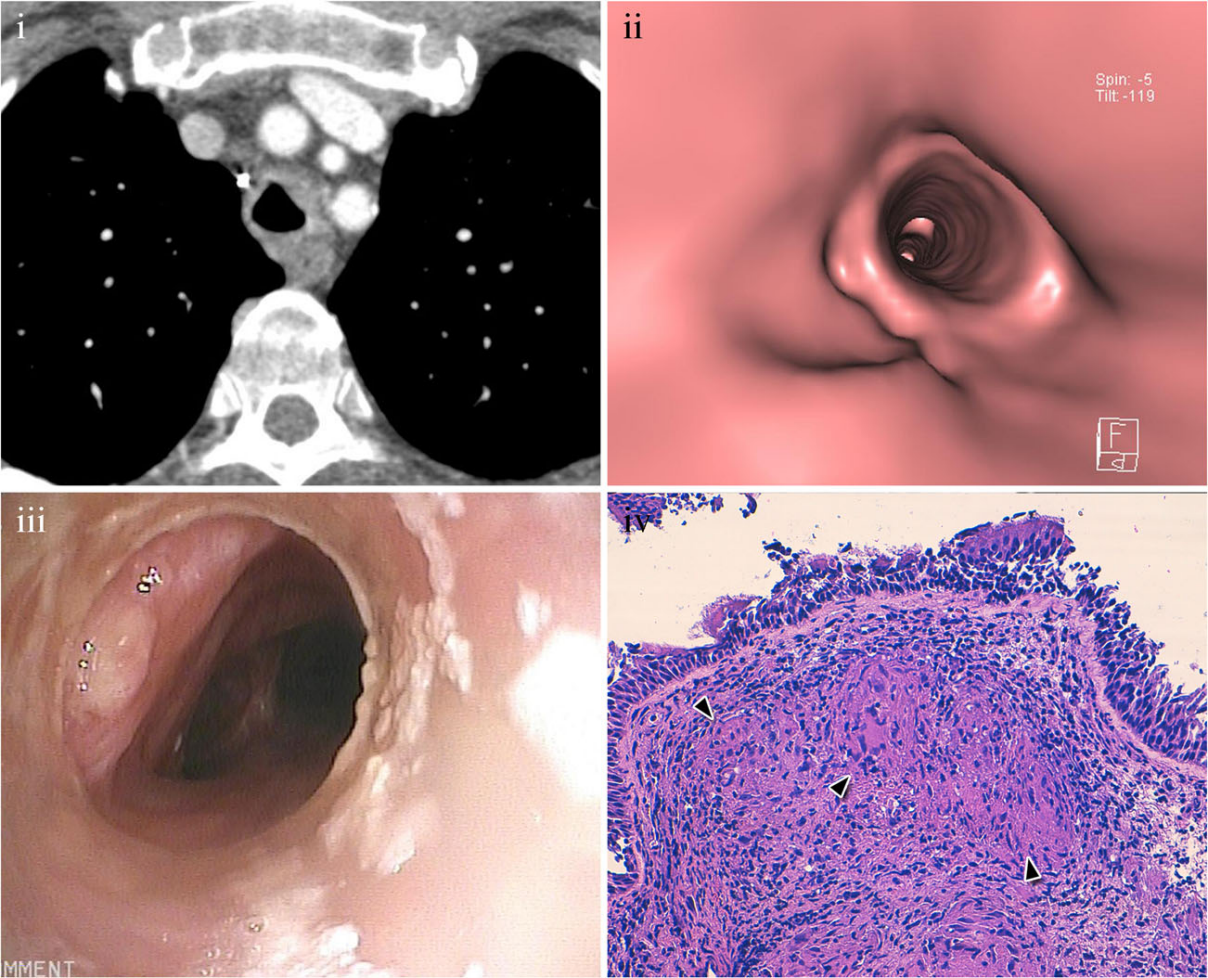
Tracheal sarcoidosis in a 59-year-old female with a history of pulmonary sarcoidosis. (**i**) Axial MDCT scan shows diffuse and irregular trachea. (**ii**) Virtual bronchoscopy and (**iii**) direct bronchoscopy have excellent correlation. (**iv**) Photomicrograph (original magnification, ×2; haematoxylin-eosin stain) shows small epithelioid granulomas without necrosis

MDCT allows suggesting the diagnosis when tracheal wall thickening and narrowing of the lumen are seen in a patient with another manifestation of sarcoidosis [24, 31] (Fig. 19).

#### Relapsing polychondritis

Relapsing polychondritis is a systemic disease, characterised by recurrent episodes of cartilaginous inflammation that lead to cartilage destruction. The disease is characterised by a chondral and perichondral inflammation secondary to an immune-mediated reaction of unknown cause, which implies antibodies against extracellular matrix components such as collagen type II and matrilin 1 [60].

This disorder affects not only the cartilaginous structures of the ears, nose, and tracheobronchial tree but also the joints, inner ear, eyes, and cardiovascular system [60].

At the time of diagnosis, the respiratory tract involves only 10% of the patients, but in the course of the disease it occurs in up to 50% [61]. Although the disease affects men and women equally, the airway affectation is more common in women [60]. Involvement of the respiratory tract carries a poor prognosis, and mortality is usually secondary to pneumonia [62].

**Fig. 20:**
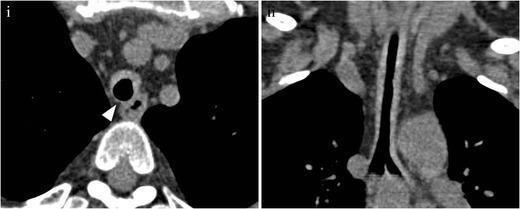
Relapsing polychondritis of the trachea in a 51-year-old patient. (**i**) Axial MDCT shows wall thickening, sparing the posterior wall of the trachea. (**ii**) Coronal MDCT shows extensive compromise of the trachea. Note the high density of the thickening wall

CT shows increased attenuation and thickening of the tracheobronchial wall, with or without mural calcifications, and sparing of the posterior membranous wall [28, 63] (Fig. 20). At a later stage, fibrosis leads to luminal irregular narrowing, and loss of the structural support of the cartilage leads to tracheobronchomalacia, which is seen as tracheobronchial collapse and air trapping on the expiratory CT scan [11, 24].

#### Tracheobronchopathia osteochondroplastica

Tracheobronchopathia osteochondroplastic is a rare idiopathic benign disorder characterised by multiple submucosal cartilaginous and osseous nodules in the respiratory tract that can involve the entire trachea and mainstem bronchi [64].

Pathogenesis of this condition is still unclear, with familial cases and others associated with chronic inflammation or trauma [65]. There is a male predilection (3:1) and it is usually diagnosed in patients over 50 years of age [66].

The bronchoscopic image is usually diagnostic, displaying multiple papilla-like protrusions from the anterior and lateral walls of the trachea, which has a cobblestone appearance, with an intact posterior wall. In advanced cases, lesions may cause airway deformity and obstruction [67]. Histologically, metaplastic cartilage and bone are found in the submucosa [68].

**Fig. 21:**
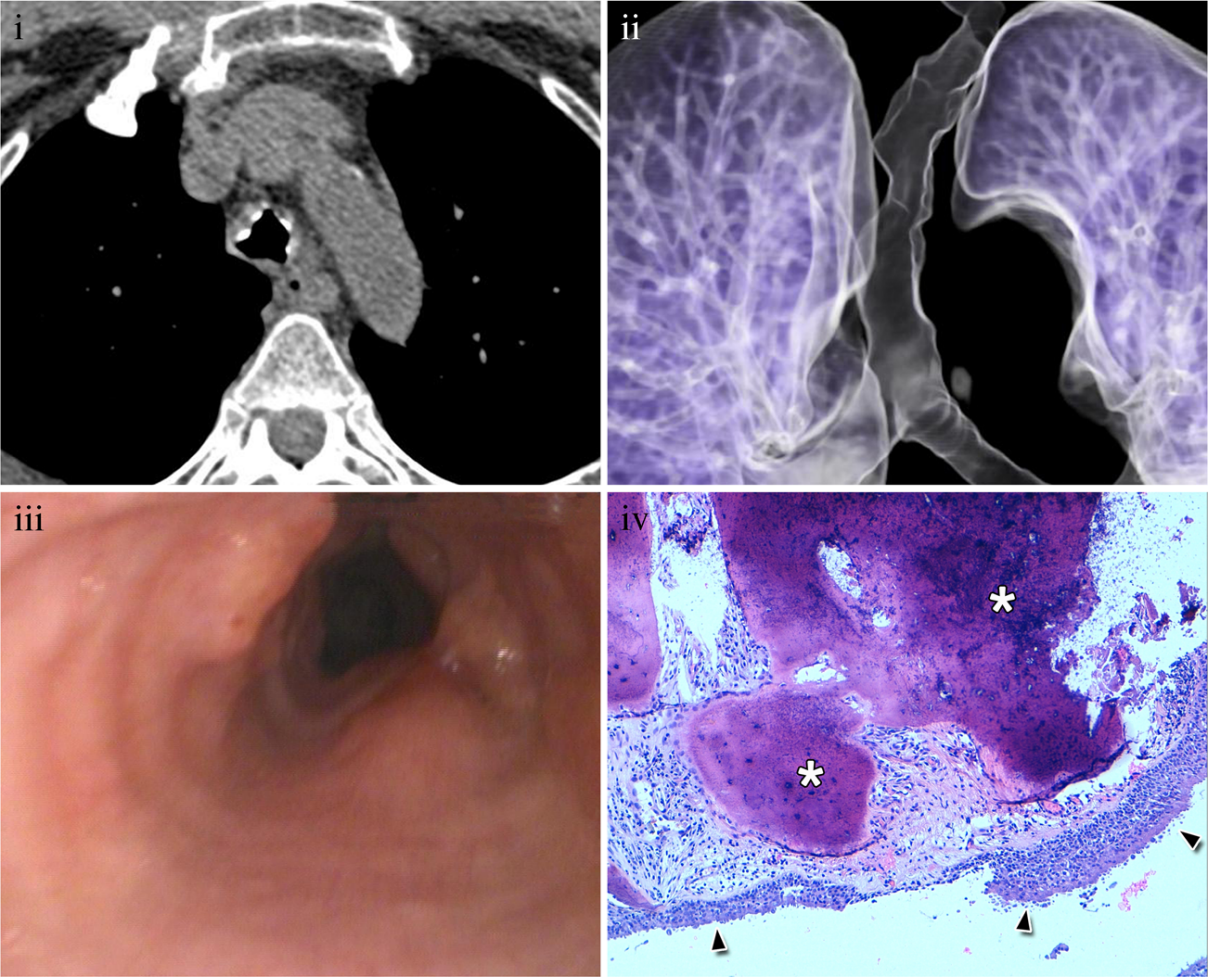
Tracheobronchopathia osteochondroplastica, as an incidental finding in a 63-year-old patient. (**i**) Axial MDCT scan shows an irregular thickening of the tracheal wall with calcifications and sparing the posterior wall. (**ii**) VRT reconstruction demonstrating the extension of the disease along the tracheal wall. (**iii**) Endoscopy demonstrated a nodular pattern without compromise of the posterior wall. (**iv**) Photomicrograph (original magnification, ×2; haematoxylin-eosin stain) shows chondroid and bony material (star) beneath the ciliated surface (*arrows*)

CT can depict thickened tracheal cartilage with irregular nodular calcification, which affects the lower two-thirds of the trachea and proximal portions of the primary bronchi [28]. Multiple nodules, with or without calcification, may project into the airway lumen, sparing the posterior membranous wall of the trachea [24, 28] (Fig. 21).

#### Tracheobronchomalacia

Tracheobronchomalacia (TBM) is characterised by an excessive expiratory airway collapse due to weakness of the airway walls secondary to alteration of the cartilaginous supports and/or redundancy of the posterior tracheal membranous wall [69].

Compromise of both the trachea and bronchi is the most common type of the disease (63%), followed by isolated tracheal involvement (22%) and, finally, isolated bronchial involvement (15%) [70].

Authors report that tracheobronchomalacia may be idiopathic, associated with prematurity and congenital cartilaginous weakness among other congenital anomalies. The acquired form is most commonly associated with prolonged mechanical endotracheal intubation, chronic airway inflammation including chronic obstructive pulmonary disease, and relapsing polychondritis [71].

Classically the diagnosis requires expiratory collapse >50%. However, recent studies conducted in healthy volunteers with normal pulmonary function have reported mean levels of expiratory collapse >50%, which would indicate the need to establish new thresholds of collapse for the diagnosis of the disease [72].

MDCT is now considered a first-line screening tool for clinically suspected TBM and may also serve as an adjunct to bronchoscopy in preoperative planning and even as an alternative to bronchoscopy in the paediatric or elderly populations [8, 9]. The acquisition technique is important to maximise the ability of the study in the diagnosis and classification of patients. At end expiration, tracheal collapse is submaximal. Therefore, dynamic acquisition during expiration must be used whenever the patient can follow instructions [73].

**Fig. 22:**
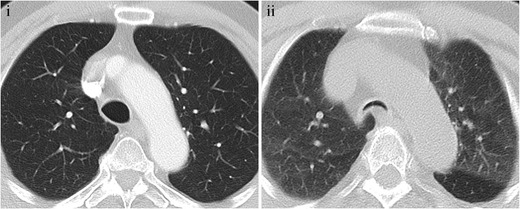
Tracheobronchomalacia in a 67-year-old patient. (**i**) Inspiratory axial MDCT shows a normal tracheal diameter. (**ii**) Expiratory axial MDCT scan demonstrating collapse of the tracheal wall during expiration, characteristic of tracheobronchomalacia

On the inspiratory MDCT scan, a lunate tracheal configuration (coronal to sagittal diameter ratio >1) is highly specific for TBM, but it has low sensitivity [74]. On the dynamic MDCT scan, a crescentic tracheal morphology, also called the “frown sign”, is highly specific for tracheobronchomalacia [74], and when it is present it may indicate the need for tracheoplasty to reinforce the posterior wall [10, 11] (Fig. 22). Concentric collapse of the tracheal lumen is less common, and it may be associated with relapsing polychondritis [74].

In patients with mucopolysaccharidosis, upper respiratory tract involvement due to glycosaminoglycan deposition in the tracheobronchial cartilages may lead to tracheal narrowing and tracheomalacia, among other causes, such as an excess of redundant parapharyngeal tissue that prolapses into the respiratory tract. It is recommended to assess the upper respiratory tract in these patients when CTs are performed for other reasons (column, craniocervical junction, etc.) [75].

#### Diagnostic approach

**Fig. 23:**
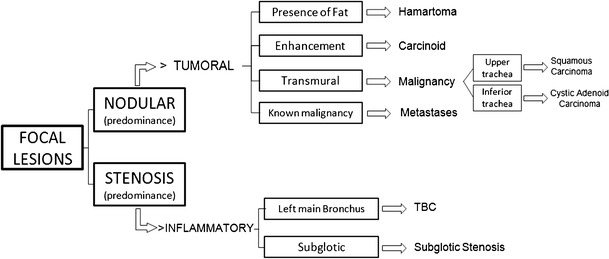
Schematic diagnostic approach to tracheobronchial focal lesions

Although most of the focal lesions are neoplastic, the distinction between nodular predominance and stenotic predominance may allow the differentiation between a neoplastic origin and non-neoplastic origin. Furthermore, the infiltrative character of a lesion is highly suggestive of malignancy (Fig. 23).

**Fig. 24:**
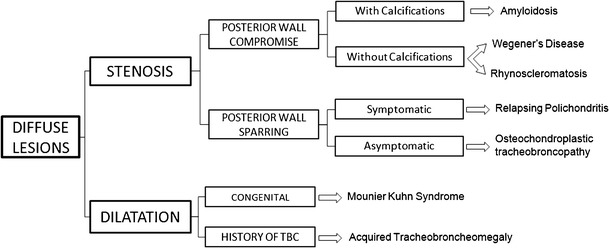
Schematic diagnostic approach of tracheobronchial diffuse lesions

Diffuse lesions have a low probability of malignancy. In these lesions, the affectation of the posterior wall and the presence of calcification may help to narrow the differential diagnosis (Fig. 24).

## Conclusion

MDCT is an excellent diagnostic method in the detection and classification of central airway pathologies. To improve MDCT’s diagnostic accuracy it is necessary to establish protocols according to clinical suspicion and use appropriate post-processing tools, such as virtual bronchoscopy.

A schematic diagnostic approach to recognise central airway disorders prevents unnecessary diagnostic tests and delays in treatment. However, a biopsy may be required to make the final diagnosis.
